# Conservative Management of Facial Talon Cusp in a Permanent Maxillary Central Incisor: A Case Report

**DOI:** 10.7759/cureus.71842

**Published:** 2024-10-19

**Authors:** Abdulelah Alqahtani, Adeeb Alnajashi

**Affiliations:** 1 Pediatric Dentistry, University Dental Hospital, King Saud University, Riyadh, SAU; 2 College of Dentistry, King Saud bin Abdulaziz University for Health Sciences, Riyadh, SAU

**Keywords:** carious lesion, dens evaginatus, dental anomaly, saudi arabia, talon cusp

## Abstract

Facial talon cusp is a rare developmental dental anomaly with unknown etiology. This case report is about an 11-year-old boy who presented with discomfort associated with tooth #11. After clinical and radiographical examination, the diagnosis of facial talon cusp (stage 1) was established. Treatment options and steps were discussed in detail. To the best of our knowledge, this is the first reported case of facial talon cusp in Saudi Arabia. The management of facial talon cusp is highly dependent on the individual patients. There are cases that only require fissure sealants; on the other hand, some cases need more aggressive treatment such as removal of the cusp, root canal treatment, or extraction. Dentists should be aware of the different treatment modalities in order to deliver the correct treatment for the patients. Clinical and radiographic examinations are important to rule out other dental anomalies. A standardized classification should be followed in classifying facial talon cusp in order to link each stage with its treatment.

## Introduction

Talon cusp (dens evaginatus) is a rare developmental dental anomaly that represents an accessory cusp-like structure associated with maxillary or mandibular anterior teeth [1]. It can be seen in both primary and permanent dentitions. The occurrence of this anomaly is reported to be more common in the male gender with permanent dentition [2]. The etiology is still unknown, but possible reasons may include the genetic or environmental contributors that affect the development of the tooth during morphodifferentiation.

The majority of cases are isolated (not associated with other syndromes), and they can be unilateral or bilateral [1]. Radiographically, it appears as a V-shaped radiopaque structure superimposed on the crown of the affected tooth. The teeth most affected by facial talon cusp are central incisors (65%), followed by lateral incisors (21%), and canines (13%) [2]. Facial talon cusp is a very rare finding. We found only 41 cases of facial talon cusp in the literature [3-33].

Hattab et al.'s classification cannot be applied because the accessory cusp is at the facial aspect of the tooth. On the other hand, Mayes, in 2007, classified facial talon cusp into three stages (see Appendices) [34]. Many complications are associated with talon cusp such as poor esthetics, irritation of soft tissues, occlusal interference, and carious lesions of deep grooves [35]. The management varies depending on the case presentation. Treatment options may include no treatment, gradual reduction, complete reduction followed by endodontic therapy and final restoration, or extraction [36]. To the best of our knowledge, this case is the first reported case of facial talon cusp in Saudi Arabia.

## Case presentation

**Figure 1:**
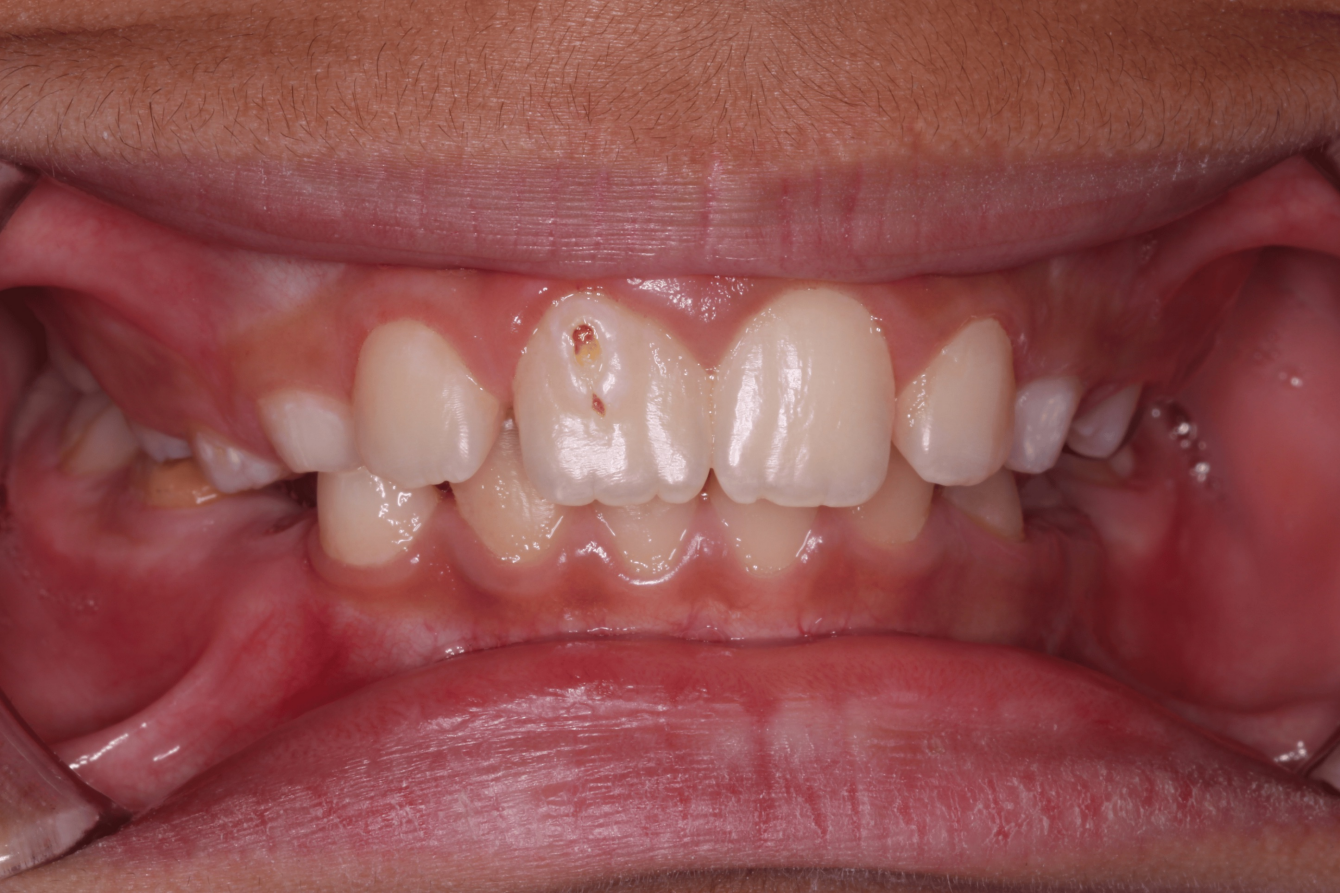
Intraoral photograph showing talon cusp on tooth #11

**Figure 2:**
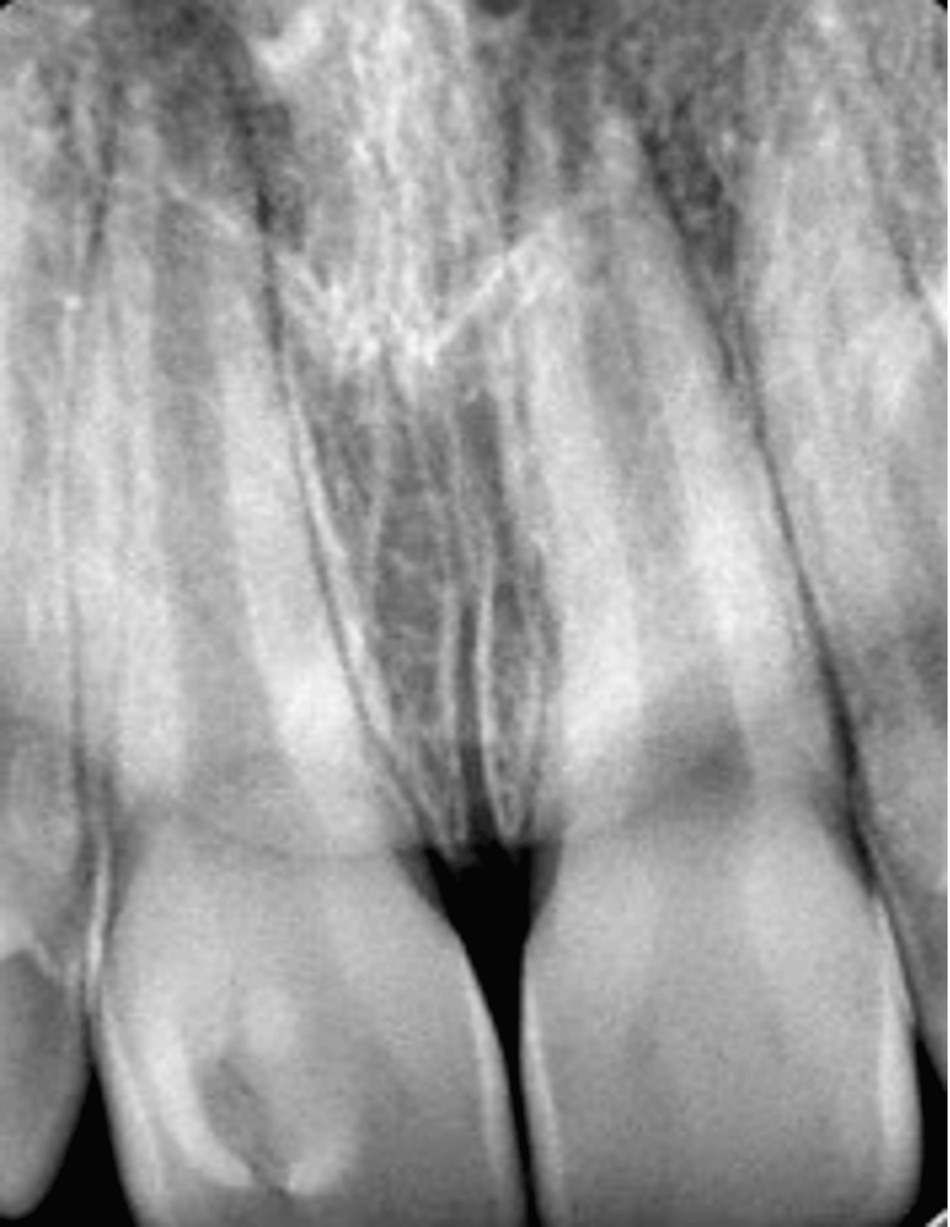
Periapical radiograph showing talon cusp on tooth #11

An 11-year-old healthy male child presented with a complaint of discomfort in the upper front tooth, pointing at the cervical area of tooth #11. Upon taking the dental history, the father who accompanied the patient did not report any history of trauma in that area. Clinical examination revealed that the patient was in the mixed dentition stage with fair oral hygiene. However, a carious lesion was found at the labial surface of tooth #11 (Figure 1) with no other carious lesions on the remaining teeth. No bleeding on probing was recorded at all teeth except for the labial sulcus of tooth #11, which is a sign of gingival inflammation. A periapical radiograph revealed the presence of pulpal tissue in the cusp (Figure 2). Moreover, the root of tooth #11 was immature with an open apex which makes maintaining the vitality of the pulp an important aspect while planning the management of this tooth. The diagnosis of facial talon cusp stage 1 was confirmed with all other teeth are normal. All treatment options were discussed with the father, and complete reduction of the cusp was the treatment of choice since they could not attend multiple appointments. 

Management

**Figure 3:**
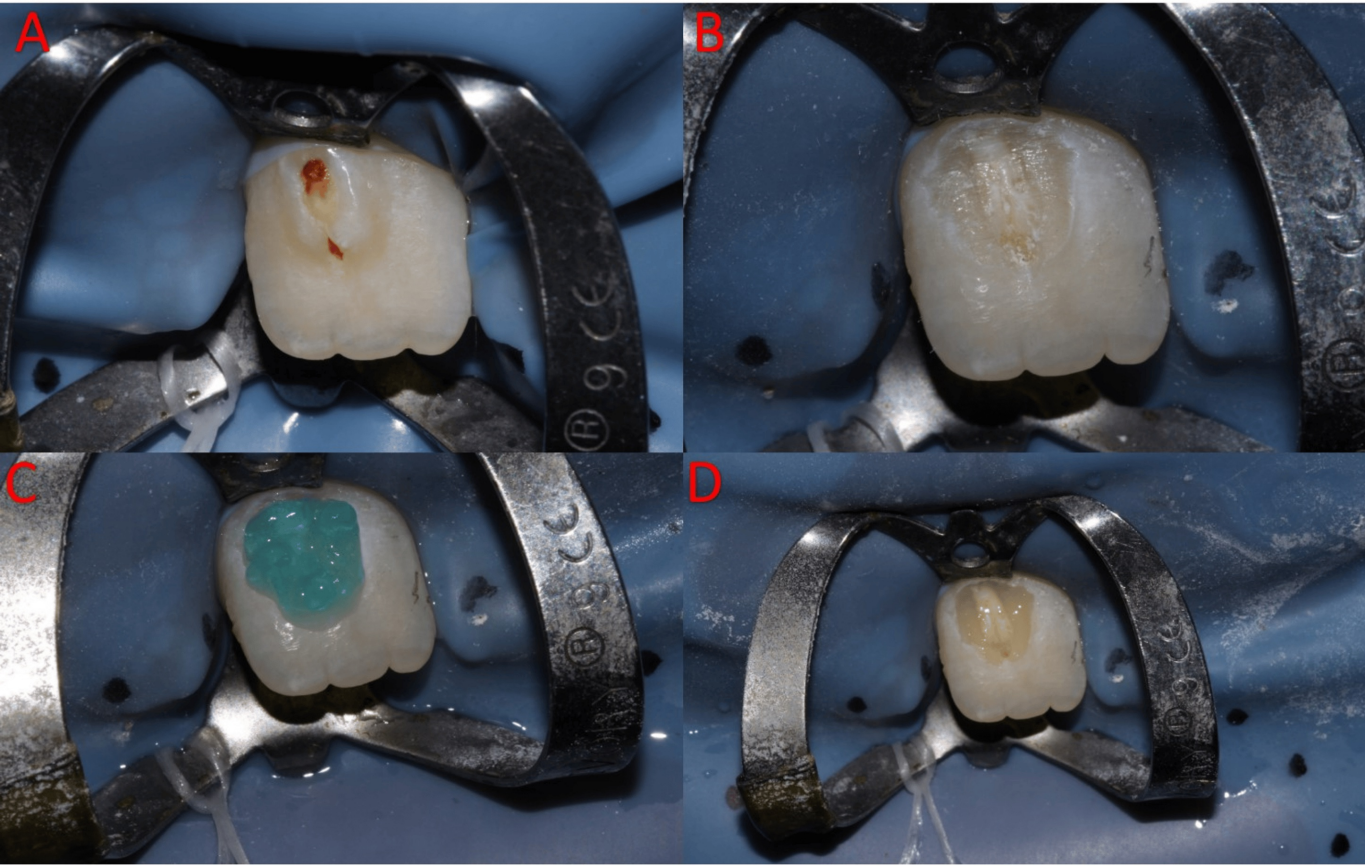
Steps of the treatment provided. (A) Clamp and rubber dam isolation. (B) Removal of the cusp. (C) Etchant application. (D) Bonding application

**Figure 4:**
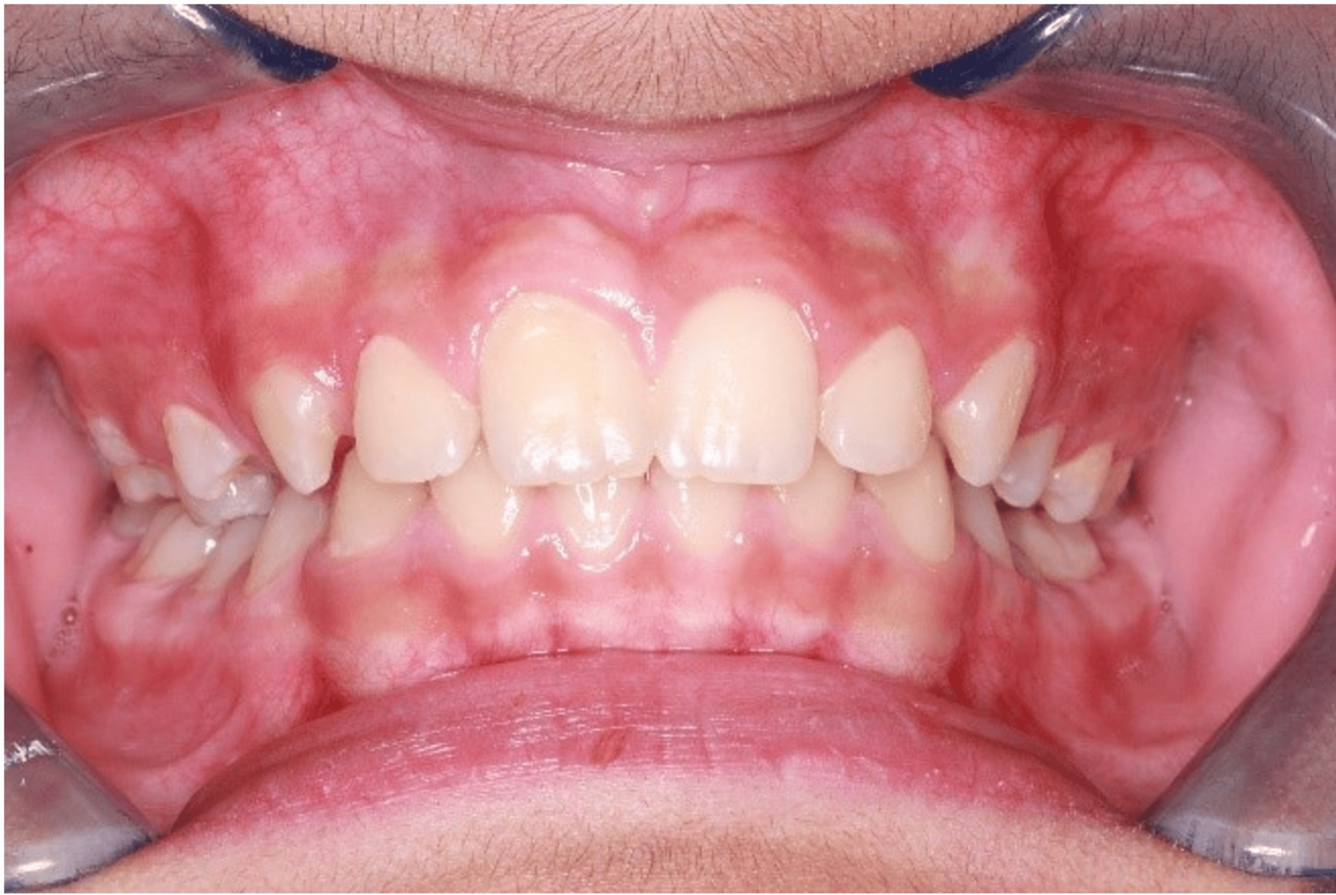
Intraoral photograph showing tooth #11 after 10 months

**Figure 5:**
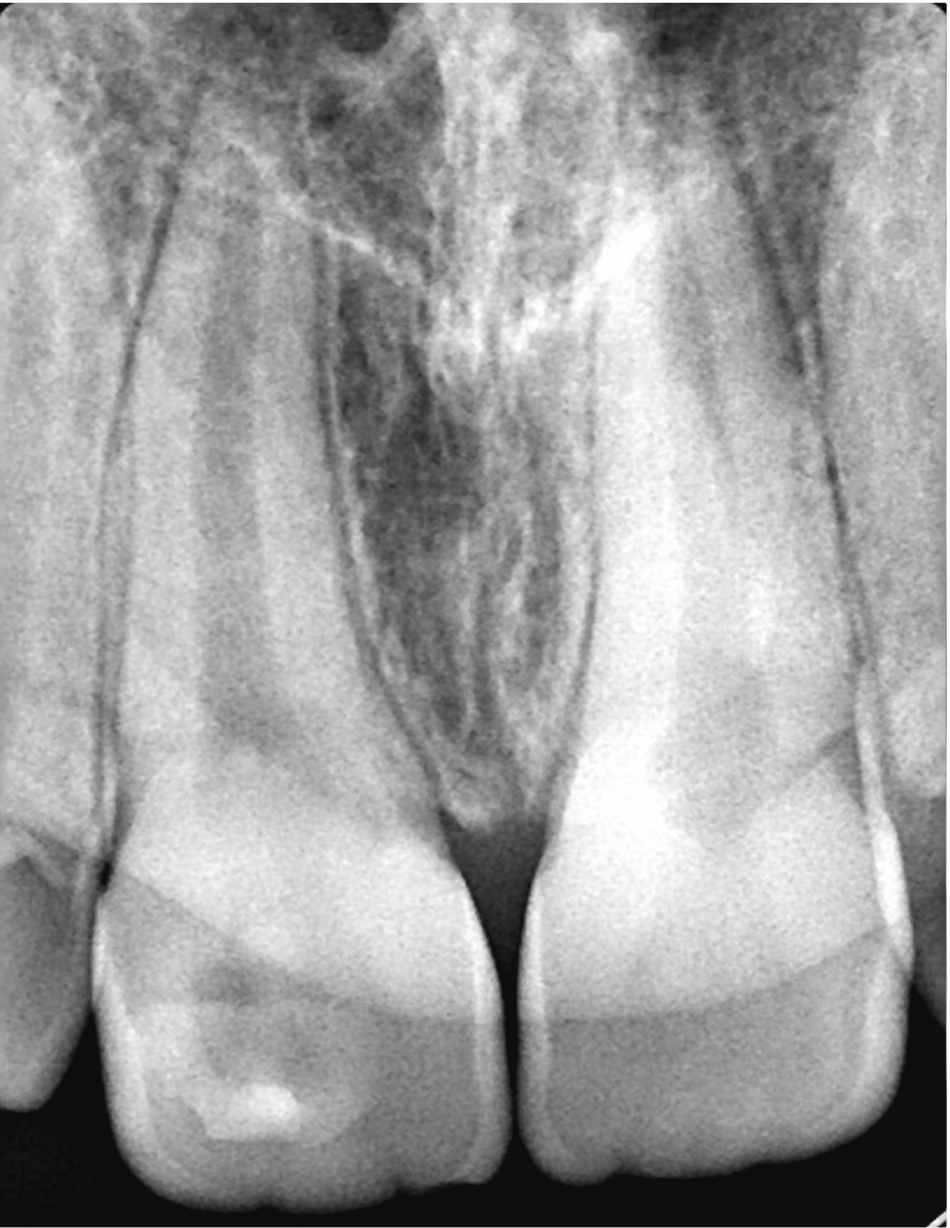
Periapical radiograph showing the closed apex of #11

Treatment started with topical anesthesia (benzocaine 20%) application and local anesthesia (lidocaine hydrochloride 2%, adrenaline 1:80000) labial infiltration of #11 and rubber dam isolation with No. 9 clamp (Figure 3A). A #330 carbide bur was used to remove the carious lesion in the cusp followed by a black round-end tapered diamond bur TF-21S (cutting) for cusp reduction, and for beveling the margins of the preparation a yellow round-end tapered diamond bur TR-25EF (finishing) (Figure 3B). No pulp exposure during the removal of the cusp. 35% phosphoric acid (Ultra-EtchTM; Ultradent Products, Inc., Utah, United States) was applied for 15 seconds and then rinsed for 10 seconds (Figure 3C). After that, 3M ESPE bonding agent (3M Company, Saint Paul, Minnesota, United States) was applied for 15 seconds followed by air thinning for five seconds then light cured for 10 seconds (Figure 3D). A class V composite restoration was done using a flowable composite (Filtek™, 3M Company) A2 shade and light cured for 20 seconds. Finishing and polishing were done using Sof-Lex™ discs (3M Company). A follow-up was done after 10 months to ensure that the apex was closed and the treatment was successful (Figures 4, 5).

## Discussion

Facial talon cusp is a rare developmental anomaly that has unknown causative factors. Race and genetics might play a major role in its occurrence. Dunn suggested that the occurrence of talon cusp on anterior teeth is more observed in Chinese and Arab populations while dens evaginatus on posterior teeth are seen more in Asian, American Indian, and Eskimo populations [12]. According to our review of the literature, this is the only case report of facial talon cusp in Saudi Arabia. We encourage dentists to report such cases to link and identify their occurrence with genetics. In one study, genetics had a major role and the presentation of the cusp observed in the patient was inherited from the father who had the same facial talon cusp [29].

Compliance from the patients is a very important factor in the treatment process. Two examples showed an obvious lack of compliance by patients which resulted in discontinuation of the treatment [3,14]. One example of incorrect management of facial talon cusp is the study of de Sousa et al., in which the first dentist just removed the cusp without restoring the tooth, which resulted in pulpal necrosis and other complications [10]. The correct management was done when the patient came at the age of 22 years, but the tooth had an immature root with an open apex, and the treatment became more complicated. Some cases do not require treatment [32,15].

In Ekambaram et al.'s study, sealant application in addition to educating the patient and parents was the required management [16]. In Prabhu et al.'s study, the cusp was asymptomatic, and the patient's concern was esthetic due to the stains in the grooves of the cusp, so enameloplasty was done to address it [30]. Gradual grinding of the cusp was done in Yazıcıoğlu and Ulukapı's case followed by a resin-based composite restoration to avoid pulp exposure [27]. On the other hand, extraction is the treatment of choice in some cases. In the case of Acharya, the facial talon cusp was on a mesiodens and extraction was the treatment [33]. Extraction in a case with an extensive presentation such as talon cusp on a geminated tooth that is affecting the eruption of adjacent teeth is justified and considered a necessity [14].

## Conclusions

Facial talon cusp is a rare developmental anomaly that might be dependent on race. All dentists are encouraged to report any similar cases in order to link and identify their occurrence with genetics. Moreover, the correct management is highly dependent on each case presentation. Dentists should be aware of the different treatment modalities in order to deliver the correct treatment for the patients. The treatment varies from simple procedures such as fissure sealants and enameloplasty to a more aggressive approach such as root canal treatment and extraction. Clinical and radiographic examinations are important to rule out other dental anomalies. A standardized classification should be followed in classifying facial talon cusp in order to link each stage with its treatment.

## Appendices

**Table 1:** Classification of facial talon cusp according to Mayes (2007) Reference: [34]

Facial talon cusp classification	
Stage 1 (slightest form)	It consists of a slightly raised triangular on the labial surface of an incisor extending the length of the crown, but not reaching the cementoenamel junction or the incisal edge
Stage 2 (moderate form)	It consists of a raised triangular on the labial surface of an incisor that extends the length of the crown, does not reach the cementoenamel junction, but does reach the incisal edge, and can be observed and palpated clearly at this stage
Stage 3 (most extreme form)	It consists of a free-form cusp extending from the cementoenamel junction to the incisal edge on the labial surface of an incisor

## References

[1] Hattab FN, Yassin OM, al-Nimri KS (1996). Talon cusp in permanent dentition associated with other dental anomalies: review of literature and reports of seven cases. ASDC J Dent Child.

[2] Hattab FN (2014). Double talon cusps on supernumerary tooth fused to maxillary central incisor: Review of literature and report of case. J Clin Exp Dent.

[3] Bommanavar S, Rajhans N, Rajput DV, Pawar VR (2022). Double facial talons on maxillary incisor-A rare case report and new proposed classification system. J Oral Maxillofac Pathol.

[4] Glavina D, Skrinjarić T (2005). Labial talon cusp on maxillary central incisors: a rare developmental dental anomaly. Coll Antropol.

[5] Schulze C (1987). Anomalies and Deformities of Human Teeth [Book in German]. Anomalien Und Missbildungen Der Menschlichen Zähne Quintessenz.

[6] Jowharji N, Noonan RG, Tylka JA (1992). An unusual case of dental anomaly: a "facial" talon cusp. ASDC J Dent Child.

[7] Abbott PV (1998). Labial and palatal “talon cusps” on the same tooth: a case report. Oral Surg Oral Med Oral Pathol Oral Radiol Endod.

[8] McKaig SJ, Shaw L (2001). Dens evaginatus on the labial surface of a central incisor: a case report. Dent Update.

[9] Turner CG (1998). Another talon cusp: what does it mean?. Dent Anthropol.

[10] de Sousa SM, Tavano SM, Bramante CM (1999). Unusual case of bilateral talon cusp associated with dens invaginatus. Int Endod J.

[11] Patil R, Singh S, Subba Reddy VV (2004). Labial talon cusp on permanent central incisor: a case report. J Indian Soc Pedod Prev Dent.

[12] Dunn WJ (2004). Unusual case of labial and lingual talon cusps. Mil Med.

[13] Oredugba FA (2005). Mandibular facial talon cusp: case report. BMC Oral Health.

[14] Cubukcu CE, Sonmez A, Gultekin V (2006). Labial and palatal talon cusps on geminated tooth associated with dental root shape abnormality: a case report. J Clin Pediatr Dent.

[15] Ma Ma, Siang M Facial talon cusp: a case report. Int Poster J Dent Oral Med.

[16] Ekambaram M, Yiu CK, King NM (2008). An unusual case of double teeth with facial and lingual talon cusps. Oral Surg Oral Med Oral Pathol Oral Radiol Endod.

[17] Hegde KV, Poonacha KS, Sujan Sujan, SG SG (2010). Bilateral labial talon cusps on permanent maxillary central incisors: report of a rare case. Acta Stomatol Croat.

[18] McNamara T, Haeussler AM, Keane J (1997). Facial talon cusps. Int J Paediatr Dent.

[19] Topaloğlu Ak A, Eden E, Ertuğrul F, Sütekin E (2008). Supernumerary primary tooth with facial and palatal talon cusps: a case report. J Dent Child (Chic).

[20] Shashikiran ND, Babaji P, Reddy VV (2005). Double facial and a lingual trace talon cusps: a case report. J Indian Soc Pedod Prev Dent.

[21] Sachdeva SK, Verma P, Dutta S, Verma KG (2014). Facial talon cusp on mandibular incisor: a rare case report with review of literature. Indian J Dent Res.

[22] Nuvvula S, Gaddam KR, Jayachandra B, Mallineni SK (2014). A rare report of mandibular facial talon cusp and its management. J Conserv Dent.

[23] Jeevarathan J, Deepti A, Muthu MS, Sivakumar N, Soujanya K (2005). Labial and lingual talon cusps of a primary lateral incisor: a case report. Pediatr Dent.

[24] Chinni S, Nanneboyina M, Ramachandran A, Chalapathikumar H (2012). A facial talon cusp on maxillary permanent central incisors. J Conserv Dent.

[25] Bhat S, Gogineni SB, Shetty SR, Fazil KA (2015). Talon cusp variations: 2 case reports. Gen Dent.

[26] Batra P, Enocson L, Hagberg C (2006). Facial talon cusp in primary maxillary lateral incisor: a report of two unusual cases. Acta Odontol Scand.

[27] Yazıcıoğlu O, Ulukapı H (2014). Management of a facial talon cusp on a maxillary permanent central incisor: a case report and review of the literature. J Esthet Restor Dent.

[28] Kulkarni VK, Choudhary P, Bansal AV, Deshmukh J, Duddu MK, Shashikiran ND (2012). Facial talon cusp: a rarity, report of a case with one year follow up and flashback on reported cases. Contemp Clin Dent.

[29] Sudhakar S, Madhavan A, Balasubramani S, Shreenivas S (2017). A rare familial presentation of facial talon cusp. J Clin Diagn Res.

[30] Prabhu R, Chatra L, Shenai P, Kishore S, Nithin S, Savitha D, Prabhu V (2014). Mandibular facial talon cusp: a rare case report. Ann Med Health Sci Res.

[31] Nandini DB, Deepak BS, Singh DN, Aparnadevi P (2021). Bilateral gemination of permanent maxillary canine with labial and palatal talon's cusps: a rare entity. J Oral Maxillofac Pathol.

[32] Hegde S, Shetty SR, Babu S (2012). The reverse claw: report of an extremely rare facial talon cusp. Dent Res J (Isfahan).

[33] Acharya S (2015). Facial talon cusp in a mesiodens: a rare occurrence. Eur J Gen Dent.

[34] Mayes AT (2007). Labial talon cusp: a case study of pre-European-contact American Indians. J Am Dent Assoc.

[35] Mellor JK, Ripa LW (1970). Talon cusp: a clinically significant anomaly. Oral Surg Oral Med Oral Pathol.

[36] Smail-Faugeron V, Picou Rollin J, Muller Bolla M, Courson F (2016). Management of non-syndromic dens evaginatus affecting permanent maxillary central incisors: a systematic review. BMJ Case Rep.

